# Case Report: When crossing is not enough: restoring functional access in an extreme brachial artery loop during primary PCI

**DOI:** 10.3389/fcvm.2026.1903487

**Published:** 2026-07-16

**Authors:** Ali Hakan Konuş, Recep Polat

**Affiliations:** Department of Cardiology, Bingöl State Hospital, Bingöl, Türkiye

**Keywords:** arterial tortuosity, bailout strategy, brachial artery loop, functional access, ST-elevation myocardial infarction, transradial intervention

## Abstract

Successful traversal of severely tortuous upper-limb arterial anatomy does not necessarily constitute functional access, yet this distinction remains poorly emphasized in the transradial literature. Existing bailout strategies primarily focus on successful traversal, whereas restoration of effective catheter mechanics through geometric correction may represent a distinct procedural challenge. We report a case of primary percutaneous coronary intervention performed via right radial access in a 73-year-old woman presenting with acute inferior ST-elevation myocardial infarction, complicated by a near-complete circular brachial artery loop. Despite successful guidewire traversal and distal advancement of a guiding catheter toward the aortic root, functional access could not be established. The underlying mechanism was not failure of traversal, but failure of geometric correction. Despite successful catheter passage, the loop persisted without meaningful straightening, resulting in inadequate catheter support and ineffective torque transmission. This case highlights the distinction between crossability — the ability to traverse a tortuous segment — and correctability — the ability of that segment to undergo geometric realignment sufficient to restore functional catheter performance. When correctability is not achieved through initial navigation, a structured escalation strategy becomes necessary. Sequential exchange to progressively higher-support guidewires, performed as protected intraluminal exchanges within the indwelling guiding catheter, ultimately achieved effective loop straightening and restored functional access. Primary PCI was completed with a door-to-balloon time of 36 minutes, demonstrating that a correctability-focused escalation strategy can be executed without compromising reperfusion efficiency in time-sensitive settings. This experience supports a mechanism-based, stepwise framework for managing extreme upper-limb arterial tortuosity — one that treats geometric correction as a distinct procedural objective and guides escalation decisions based on functional access assessment rather than traversal success alone. This reframing offers a practical framework for recognizing when successful traversal alone is insufficient to establish functional access, for guiding structured escalation, and for informing more deliberate access-site crossover decisions in acute revascularization settings.

## Introduction

Transradial access has become the preferred approach for coronary angiography and percutaneous coronary intervention (PCI), supported by robust evidence demonstrating reductions in vascular complications, major bleeding, and mortality compared with the transfemoral route (1–3). Its adoption in acute coronary syndromes has been further endorsed by contemporary guidelines, reflecting consistent findings from large randomized trials (4). However, procedural success depends not only on obtaining arterial access, but on the ability to effectively utilize the upper-limb arterial axis as a delivery conduit. Extreme tortuosity or loop configurations of the upper-limb vasculature represent a clinically relevant source of procedural difficulty, and their management remains incompletely defined (5–7).

We report a case of primary PCI performed via right radial access in a patient with acute inferior ST-elevation myocardial infarction (STEMI), complicated by extreme upper-limb arterial tortuosity with a near-complete circular (O-shaped) brachial artery loop. Despite successful wire crossing and distal advancement of a guiding catheter, adequate catheter support for coronary engagement could not be achieved, underscoring the distinction between successful traversal and effective catheter performance. This case illustrates the distinction between successful traversal and functional access, and proposes a structured, correctability-oriented framework for managing extreme upper-limb tortuosity in time-sensitive settings.

## Case presentation

A 73-year-old woman (weight 56 kg) with a history of type 2 diabetes mellitus, hypertension, and known coronary artery disease (prior anterior myocardial infarction treated with left anterior descending artery stenting) presented to the emergency department with typical chest pain. Electrocardiography showed ST-segment elevation in the inferior leads, consistent with acute inferior myocardial infarction. The patient was taken emergently to the catheterization laboratory for primary PCI.

**Figure 1:**
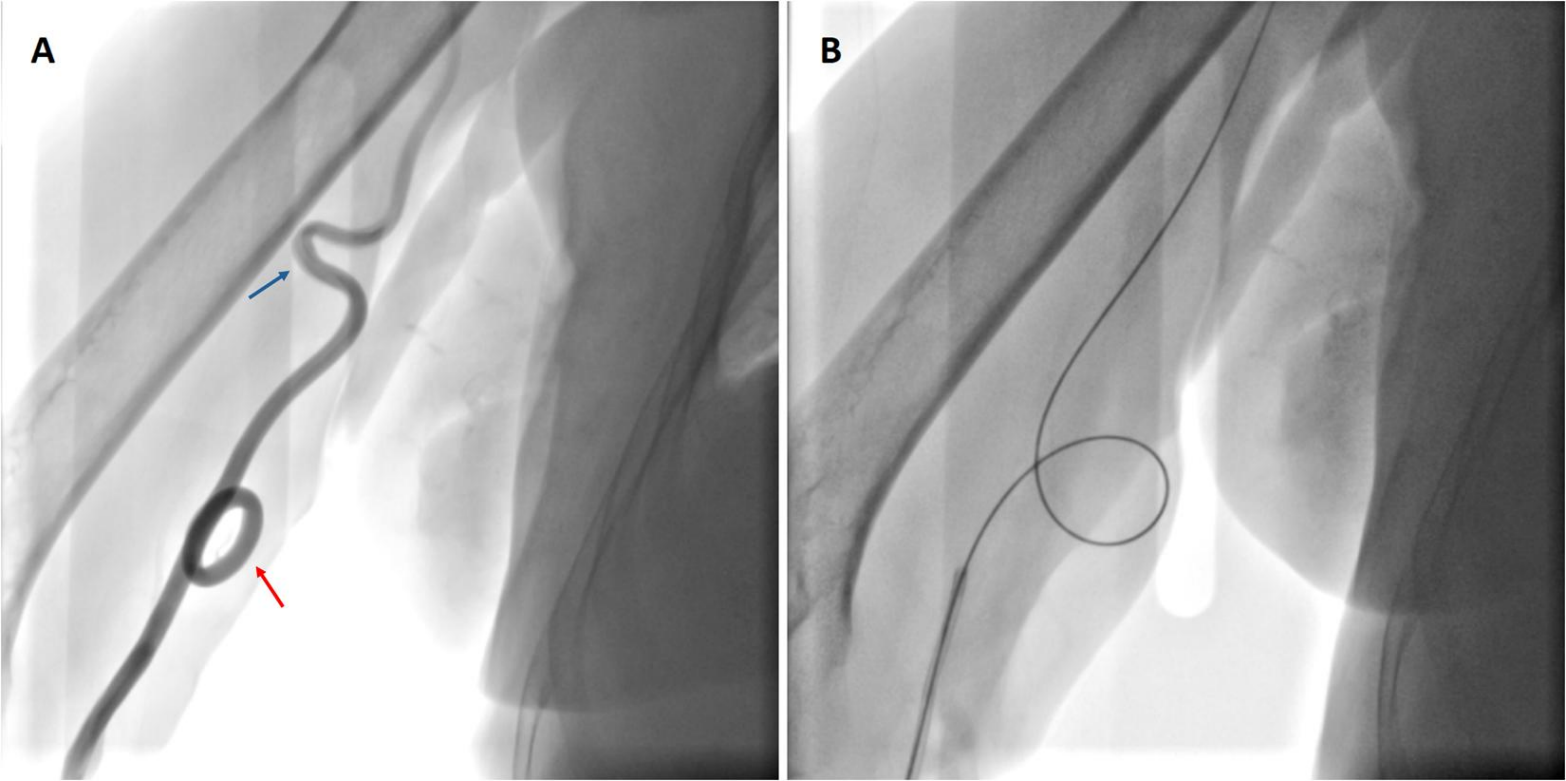
Near-complete circular brachial artery loop and failure of hydrophilic wire–mediated geometric correction. **(A)** Angiographic image of the right upper-limb arterial axis demonstrating a near-complete circular (O-shaped) loop of the brachial artery (red arrow), with reversal of the vessel trajectory and additional proximal tortuosity (blue arrow). **(B)** Advancement of a 0.035-inch hydrophilic guidewire across the brachial artery loop showing persistence of the loop morphology without effective geometric correction.

Preprocedural assessment confirmed bilateral radial artery patency by a modified Allen test. Right radial artery access was obtained using a 6-French sheath. Dual antiplatelet therapy was initiated in the emergency department with loading doses of aspirin 300 mg and ticagrelor 180 mg orally. Anticoagulation consisted of unfractionated heparin 5,000 units administered intravenously in the emergency department, with an additional 1,000 units delivered via the radial cocktail, which also included nitroglycerin 200 mcg for vasospasm prophylaxis. During advancement of the guidewire, significant resistance was encountered at the level of the upper limb. Angiography of the right upper-limb arterial axis revealed marked elongation with a near-complete circular (O-shaped) loop of the brachial artery in the distal upper arm, confirmed fluoroscopically with reference to osseous landmarks, with additional proximal tortuosity (Figure 1A).

**Figure 2:**
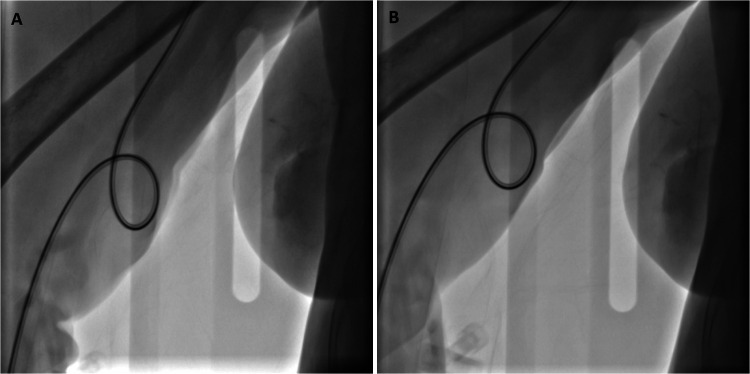
Failure of standard support strategies to achieve functional access in the presence of a brachial artery loop. **(A)** Advancement of a Judkins Right guiding catheter over a hydrophilic guidewire demonstrating persistence of the brachial artery loop despite distal catheter advancement. **(B)** Exchange to a standard non-hydrophilic 0.035-inch guidewire failing to achieve geometric straightening, with the brachial artery loop remaining unchanged.

A 0.035-inch hydrophilic guidewire was advanced across the brachial artery loop; however, despite successful traversal, no meaningful geometric correction was achieved (Figure 1B). A Judkins Right (JR) guiding catheter was advanced over the wire and positioned distally toward the aortic root. However, the brachial artery loop remained uncorrected, and the guiding catheter could not be manipulated with adequate support for coronary engagement (Figure 2A).

The hydrophilic guidewire was subsequently exchanged for a standard non-hydrophilic 0.035-inch guidewire within the guiding catheter to improve axial support. Despite this modification, the brachial artery loop persisted without effective geometric straightening (Figure 2B).

**Figure 3:**
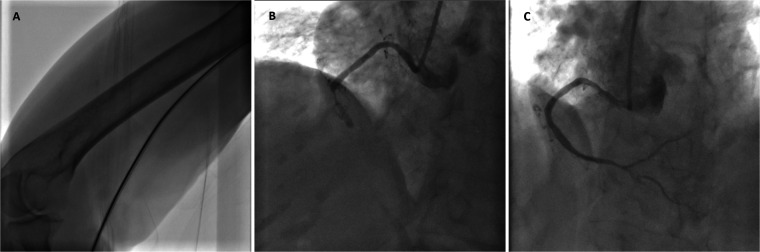
Restoration of functional access with high-support wire and completion of primary PCI. **(A)** Advancement of an Amplatz Super Stiff guidewire resulting in effective geometric straightening of the brachial artery and restoration of a near-linear vessel course. **(B)** Coronary angiography demonstrating total occlusion of the right coronary artery as the culprit lesion. **(C)** Final angiographic result after successful stent implantation in the right coronary artery showing restoration of TIMI 3 flow. PCI, percutaneous coronary intervention; TIMI, Thrombolysis In Myocardial Infarction.

Given the persistent mechanical limitation, escalation to a higher-support system was undertaken. An Amplatz Super Stiff 0.035-inch guidewire was introduced within the guiding catheter as a protected intraluminal exchange. With controlled manipulation under continuous fluoroscopic guidance, the brachial artery loop was effectively straightened, restoring a near-linear vessel course (Figure 3A). This maneuver enabled effective and stable manipulation of the guiding catheter with adequate support for coronary engagement. Following confirmation of loop straightening, the Amplatz Superstiff guidewire was withdrawn while the Judkins Right guiding catheter was maintained in position. The restored geometric alignment was sustained by the indwelling catheter, which was subsequently advanced to engage the right coronary artery ostium for coronary intervention.

Coronary angiography demonstrated total occlusion of the right coronary artery as the culprit lesion (Figure 3B). Primary PCI was successfully performed with implantation of two drug-eluting stents in the mid and distal right coronary artery (3.0 × 26 mm and 3.5 × 18 mm, respectively). Post-dilation of the stent overlap zone was performed using a non-compliant balloon to optimize stent apposition. TIMI 3 flow was restored (Figure 3C). Adequate anticoagulation was confirmed with an activated clotting time of 278 seconds prior to intervention. A door-to-balloon time of 36 minutes was achieved despite the anatomical complexity encountered.

No access-site or peripheral vascular complications were observed during or after the procedure. Prior to discharge, duplex ultrasonography of the right upper limb confirmed normal triphasic flow patterns in both the radial and brachial arteries, with no evidence of dissection, thrombosis, or hemodynamically significant vascular injury at the access site or at the level of the brachial artery loop.

## Discussion

Transradial access is now the recommended default strategy for coronary intervention in acute coronary syndromes, offering well-established reductions in bleeding, vascular complications, and mortality compared with the femoral approach (1–3). However, procedural success depends not only on vascular access but also on the ability to traverse and functionally utilize the upper-limb arterial axis as an effective delivery conduit. In this context, the literature has primarily focused on the technical challenge of advancing a guidewire or catheter through tortuous segments — a property that may be termed crossability. In contrast, less attention has been given to a distinct but equally critical property: correctability — defined here as the capacity of a tortuous segment to undergo geometric realignment sufficient to restore functional catheter mechanics. Although operators routinely assess the ease of wire or catheter passage through tortuous anatomy, the distinction between successful traversal and restoration of functional access has received little explicit attention in the transradial literature. The concepts of crossability and correctability may provide a useful framework for describing these complementary but distinct procedural properties. The present case illustrates the practical implications of this distinction, demonstrating that successful wire traversal and catheter advancement alone may be insufficient to establish functional access in extreme loop configurations, particularly in the absence of effective geometric correction. One possible explanation for this imbalance is that successful traversal produces an immediately observable fluoroscopic result, whereas restoration of functional access depends on catheter behavior, torque transmission, and procedural performance — parameters that are assessed indirectly and often intuitively by experienced operators. As a result, correctability may be recognized in practice but has been less frequently conceptualized as a distinct procedural property warranting explicit assessment.

Loop configurations of the upper-limb arterial axis represent a recognized source of procedural difficulty during transradial intervention. While radial loops have been reported in approximately 1–2% of procedures and are associated with lower procedural success rates even among experienced operators (5, 6, 8), brachial artery loops may present a distinct mechanical challenge, particularly when extreme in configuration. Unlike radial loops, which are typically located in a distal, smaller-caliber segment, brachial loops involve a larger-caliber, more proximal vessel with greater elastic recoil, rendering geometric correction more demanding and increasing the importance of controlled, safety-oriented manipulation. The prevalence of brachial artery loops as a distinct anatomical entity remains incompletely characterized in the literature, with limited representation in transradial procedural registries. In the present case, the loop was localized to the brachial artery and confirmed fluoroscopically in conjunction with osseous landmarks of the distal upper arm. The loop configuration represented not merely a navigational obstacle but a structural impediment to effective catheter mechanics. This configuration was not adequately resolved by wire traversal or catheter advancement alone. Against this background, functional access was not defined solely by successful traversal, but by the ability to advance and manipulate the guiding catheter with stable positioning, effective torque transmission, and sufficient backup support to achieve coronary engagement and device delivery. By this definition, functional access was not established despite successful traversal and catheter advancement, underscoring that crossability and correctability represent distinct but interdependent procedural challenges in extreme loop configurations.

The sequential escalation of support in this case followed a deliberate, mechanism-driven rationale. Hydrophilic guidewires are well-suited for navigating tortuous segments owing to their low surface friction and enhanced trackability; however, their inherent flexibility limits their capacity to generate the axial force required for geometric realignment (9). In the present case, a 0.035-inch hydrophilic guidewire was used, though small-caliber hydrophilic coronary wires may represent an alternative in selected anatomical contexts, particularly when vessel caliber or loop morphology precludes advancement of larger-caliber systems (9). Accordingly, successful traversal of the loop with a 0.035-inch hydrophilic wire — while an important first step — did not translate into effective geometric correction in the present case. Advancement of a simple-curve guiding catheter over the hydrophilic wire facilitated atraumatic passage through the loop and established a protective intraluminal conduit for subsequent wire exchanges. However, persistent elastic recoil prevented effective geometric realignment despite successful catheter advancement. Subsequent exchange to a standard non-hydrophilic guidewire, performed as a protected intraluminal exchange within the guiding catheter, provided greater axial stiffness but remained insufficient to remodel the loop geometry. In clinical practice, this intermediate escalation step may encompass a range of non-hydrophilic guidewires with progressively greater shaft support, reflecting differences in operator preference, anatomical considerations, and device availability. Despite successful traversal and catheter positioning, functional access remained inadequate at this stage, as effective catheter mechanics could not be established. This indicated that correctability had not yet been achieved and justified further escalation. These sequential findings indicate that escalation decisions should not be guided solely by successful passage of a wire or catheter through the tortuous segment, but by repeated assessment of geometric correction and restoration of functional access. High-support guidewires have an established role in facilitating catheter delivery through tortuous upper-limb anatomy. High-support systems represent a distinct escalation stage; examples include the Amplatz Super Stiff, Lunderquist, and other high-support guidewires capable of transmitting sufficient axial force to facilitate geometric realignment of extreme loop configurations. The present report builds upon this existing experience by incorporating such devices into a structured, correctability-oriented framework, in which escalation is guided by progressive geometric correction and restoration of functional access following successful traversal. Escalation to an Amplatz Superstiff guidewire, similarly introduced as a protected intraluminal exchange, ultimately provided sufficient structural support to achieve effective loop straightening with restoration of a near-linear vessel course.

**Figure 4:**
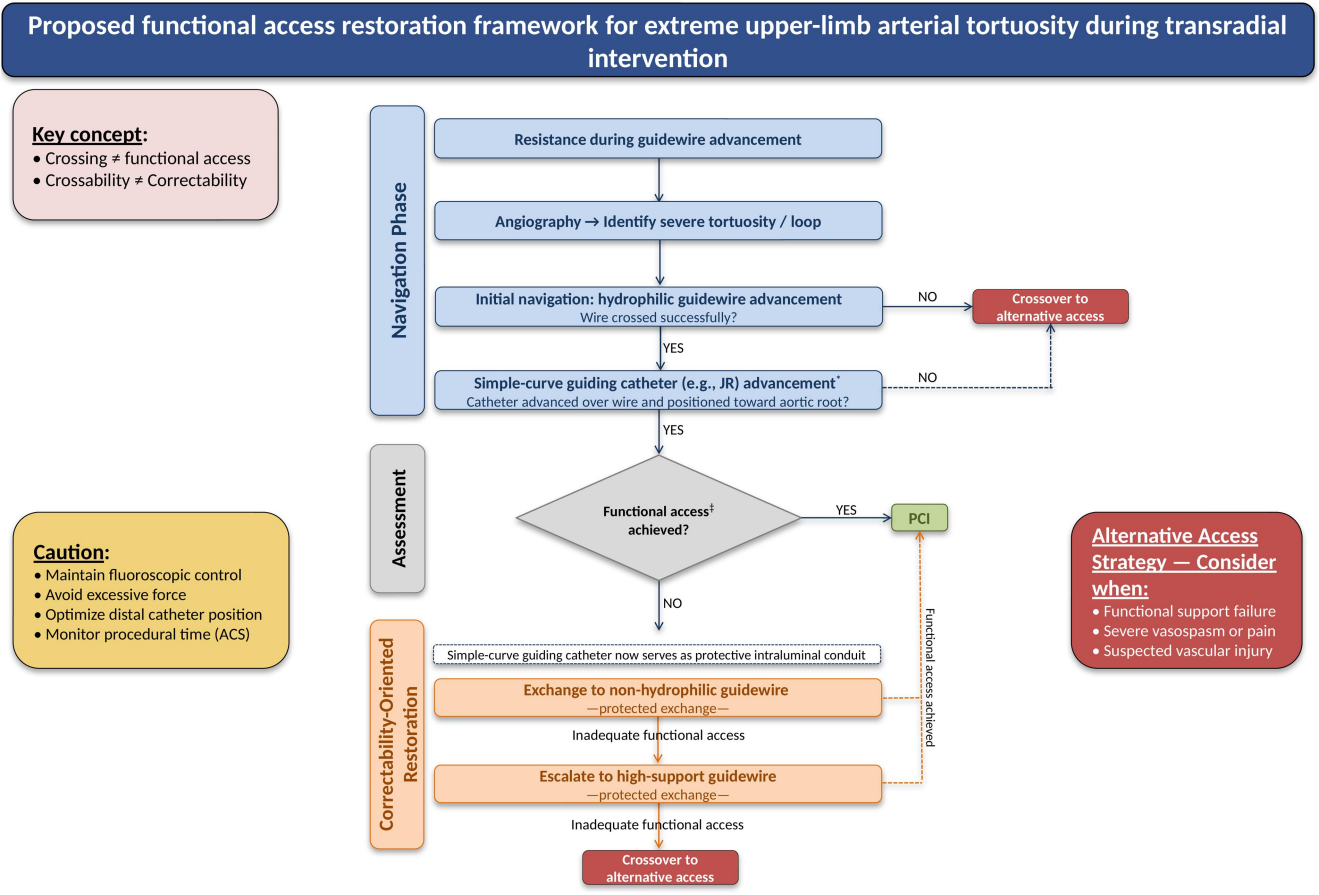
Proposed functional access restoration framework for extreme upper-limb arterial tortuosity during transradial intervention. The framework is triggered by resistance during guidewire advancement, prompting angiographic identification of severe tortuosity or loop configuration. The navigation phase encompasses initial crossing with a hydrophilic guidewire and subsequent advancement of a simple-curve guiding catheter toward the aortic root. Successful completion of these steps does not, in itself, constitute functional access. Functional access requires sufficient geometric correction to restore effective catheter mechanics. This property (correctability) is distinct from the ability to traverse the segment (crossability). If functional access cannot be confirmed following initial navigation, a correctability-oriented escalation strategy is undertaken, consisting of sequential exchange to progressively higher-support guidewires performed as protected intraluminal exchanges within the indwelling guiding catheter. Crossover to an alternative access site is recommended if functional access cannot be established despite escalation, or in the presence of complications such as severe vasospasm, pain, or suspected vascular injury. Not all escalation steps are required in every case; progression is guided by geometric correction and functional access assessment at each stage. *A range of adjunctive techniques — including balloon-assisted tracking, mother-child catheter systems, low-profile or sheathless guiding systems, small-caliber coronary guidewires, and other catheter delivery strategies — may facilitate catheter advancement when initial passage cannot be achieved. Techniques that augment backup support may play a secondary role in extreme loop configurations where geometric realignment remains the primary procedural objective. ^‡^Functional access is defined as the ability to advance and manipulate the guiding catheter with stable positioning, effective torque transmission, and sufficient backup support to enable coronary engagement and device delivery. ACS, acute coronary syndrome; JR, Judkins Right; PCI, percutaneous coronary intervention.

Previously described transradial strategies for tortuous anatomy have largely focused on facilitating guidewire passage and catheter advancement across anatomically challenging segments. By contrast, explicit consideration of restoration of functional access as a procedural objective distinct from successful traversal appears to be limited in the published transradial literature. The present framework explicitly treats restoration of functional access as a separate procedural objective and uses geometric correctability to guide escalation decisions following successful traversal. In the present case, escalation was triggered not by inability to advance the guidewire or catheter, but by persistence of inadequate catheter mechanics despite successful distal catheter positioning. This stepwise progression aligns with prior reports in which a range of techniques — including balloon-assisted tracking, mother-child catheter systems, low-profile or sheathless guiding systems, and small-caliber coronary guidewires — have been used to address catheter delivery challenges and augment support during transradial intervention (9–11). These approaches primarily facilitate catheter navigation or enhance backup support across tortuous segments. However, in extreme loop configurations, successful catheter passage alone may be insufficient to establish functional access, and geometric correction may represent a distinct procedural objective not consistently achievable through these techniques alone. Among these methods, mother-child catheter systems have an established role in facilitating device delivery through resistant or tortuous coronary segments (10), and may additionally augment backup support when functional access remains inadequate; however, their optimal timing in the context of peripheral arterial tortuosity, and their contribution to geometric correction in such settings, remain less well defined. When wire and catheter navigation can be achieved without significant resistance, adjunctive catheter support techniques may be deferred in favor of a direct transition to a stepwise correction strategy. The present case integrates these principles into a safety-oriented framework in which restoration of functional access, rather than successful traversal alone, serves as the primary procedural objective (Figure 4).

The simple-curve guiding catheter appeared to serve multiple and phase-dependent roles in this case. During the navigation phase, its soft-tip configuration facilitated atraumatic traversal of the loop. Advancement of the catheter over the hydrophilic wire may itself contribute to partial geometric modification, although this effect is not consistent in extreme loop configurations and was not sufficient to establish functional access in the present case. During the escalation phase, the guiding catheter may assume a distinct role as a protective intraluminal conduit, enabling coaxial exchange to progressively stiffer guidewires beyond the loop while minimizing direct vessel–wire interaction — a critical safety consideration when advancing high-support systems through severely tortuous anatomy. Advancing the catheter toward the aortic root may serve both functional and safety purposes: it is a prerequisite for meaningful assessment of torque transmission and backup support, and reduces the length of tortuous vessel exposed to direct wire interaction during subsequent high-support exchanges. Collectively, these phase-dependent roles suggest that the simple-curve guiding catheter may function not only as a passive conduit but also as an active component of the correctability strategy.

The clinical context of acute inferior STEMI introduced additional time-sensitive procedural complexity. Current guidelines recommend a door-to-balloon time of less than 90 minutes, and even short delays in reperfusion may carry clinically relevant prognostic implications (12). In this setting, access crossover — while remaining a valid and often necessary option — is not a neutral decision. Although crossover — whether to the contralateral radial or femoral artery — may introduce modest delays at the population level, its impact in individual high-risk patients may still be clinically meaningful. More importantly, crossover to the femoral artery may forfeit the well-established benefits of transradial access, including lower rates of major bleeding and improved clinical outcomes compared with the transfemoral approach, particularly in elderly patients receiving antithrombotic therapy (2, 3). Against this background, the present case suggests that a structured, stepwise escalation strategy may allow extreme upper-limb tortuosity to be addressed without compromising reperfusion efficiency. A door-to-balloon time of 36 minutes — well within guideline recommendations and achieved despite the anatomical complexity encountered — supports the feasibility of maintaining a transradial approach in time-sensitive settings, provided that escalation steps are performed rapidly and decisively. This efficiency reflects a strategy in which each step is actively evaluated for geometric correction and progression toward functional access, rather than continued despite limited functional improvement.

This safety-oriented approach is particularly relevant when escalating to high-support systems. Attempts to achieve geometric correction carry a theoretical risk of vessel trauma, including arterial dissection, perforation, vasospasm, or endothelial injury, particularly when high-support systems are advanced without adequate imaging guidance or through forceful manipulation. To mitigate these risks, high-support wires should ideally be used under continuous fluoroscopic guidance, with geometric realignment achieved through controlled force transmission rather than forceful manipulation. In the present case, the Amplatz Superstiff guidewire was advanced with the wire tip positioned in the ascending aorta under continuous fluoroscopic monitoring, with vessel straightening achieved through controlled manipulation rather than aggressive advancement. All escalation steps were performed as protected intraluminal exchanges within the guiding catheter, reducing direct vessel–wire interaction during high-support manipulation. These measures collectively reflect a strategy in which procedural safety and geometric correction are pursued in parallel rather than competing priorities.

This report is inherently limited by its single-case design and does not permit estimation of the prevalence of this anatomical variant or comparison with alternative strategies. The absence of a comparative framework precludes definitive conclusions regarding the superiority of the described approach, and the proposed concepts of crossability and correctability should be regarded as hypothesis-generating, requiring validation in larger case series and prospective studies. Functional access was assessed pragmatically based on operator judgment supported by procedural performance surrogates — including catheter stability, torque transmission, and effective device delivery — rather than predefined quantitative criteria. The limited literature on brachial artery loop morphology further constrains direct comparison with previously reported techniques. In addition, long-term vascular outcomes could not be assessed, and the reproducibility of this approach across different operators and anatomical variations remains uncertain. A key strength of this case lies in the sequential angiographic documentation of each procedural step. This provides mechanistic insight into the distinction between successful traversal and functional access, and illustrates the potential role of structured geometric correction in extreme upper-limb tortuosity.

## Conclusion

This case illustrates that successful traversal of a severely tortuous upper-limb arterial segment does not necessarily constitute functional access. The distinction between crossability and correctability provides a clinically relevant framework for procedural decision-making in extreme loop configurations, highlighting that geometric correction, rather than successful traversal alone, may represent a key determinant of functional catheter performance. When correctability is not achieved through initial navigation, a structured, correctability-oriented escalation strategy may restore functional access without compromising reperfusion efficiency, as reflected by the door-to-balloon time achieved in the present case, and may help define the appropriate timing of access-site crossover when escalation proves unsuccessful. Further validation in larger case series and prospective studies is needed to establish the generalizability of this framework across different anatomical scenarios and operator settings.

## Data Availability

The original contributions presented in the study are included in the article/Supplementary Material, further inquiries can be directed to the corresponding author.

## References

[1] Jolly SS Yusuf S Cairns J Niemelä K Xavier D Widimsky P. Radial versus femoral access for coronary angiography and intervention in patients with acute coronary syndromes (RIVAL): a randomised, parallel group, multicentre trial. Lancet. (2011) 377(9775):1409–1420. 10.1016/S0140-6736(11)60404-2 21470671

[2] Valgimigli M Gagnor A Calabró P Frigoli E Leonardi S Zaro T. Radial versus femoral access in patients with acute coronary syndromes undergoing invasive management: a randomised multicentre trial. Lancet. (2015) 385(9986):2465–2476. 10.1016/S0140-6736(15)60292-6 25791214

[3] Gargiulo G Giacoppo D Jolly SS Cairns J Le May M Bernat I. Effects on mortality and major bleeding of radial versus femoral artery access for coronary angiography or percutaneous coronary intervention: meta-analysis of individual patient data from 7 multicenter randomized clinical trials. Circulation. (2022) 146(18):1329–1343. 10.1161/CIRCULATIONAHA.122.061527 36036610

[4] Colvin MM Cook JL Chang PP Hsu DT Kiernan MS Kobashigawa JA. An update on radial artery access and best practices for transradial coronary angiography and intervention in acute coronary syndrome: a scientific statement from the American Heart Association. Circ Cardiovasc Interv. (2018) 11(9):e000035. 10.1161/CIR.0000000000000598 30354598

[5] Garg N Sagar P Kapoor A. Arterial anomalies of the upper limb and their influence on transradial coronary procedures. J Invasive Cardiol. (2021) 33(3):E165–E171. 33542160

[6] Lo TS Nolan J Fountzopoulos E Behan M Butler R Hetherington SL. Radial artery anomaly and its influence on transradial coronary procedural outcome. Heart. (2009) 95(5):410–415. 10.1136/hrt.2008.150474 18977799

[7] Valsecchi O Vassileva A Musumeci G Rossini R Tespili M Guagliumi G. Failure of transradial approach during coronary interventions: anatomic considerations. Catheter Cardiovasc Interv. (2006) 67(6):870–878. 10.1002/ccd.20732 16649233

[8] Louvard Y Lefevre T Morice MC. Loops and transradial approach in coronary diagnosis and intervention. Cathet Cardiovasc Diagn. (2000) 51(3):250–253. 10.1002/1522-726x(200010)51:2<250::aid-ccd24>3.0.co;2-0 10.1002/1522-726x(200010)51:2<250::aid-ccd24>3.0.co;2-0 11025586

[9] Patel T Shah S Pancholy S Rao S Bertrand OF Kwan T. Balloon-assisted tracking: a must-know technique to overcome difficult anatomy during transradial approach. Catheter Cardiovasc Interv. (2014) 83(2):211–220. 10.1002/ccd.24959 23592578

[10] Takeshita S Shishido K Sugitatsu K Okamura N Mizuno S Yaginuma K. *In vitro* and human studies of a 4F double-coaxial technique (“mother-child” configuration) to facilitate stent implantation in resistant coronary vessels. Circ Cardiovasc Interv. (2011) 4(2):155–161. 10.1161/CIRCINTERVENTIONS.110.957290 21364150

[11] Gilchrist IC. Eliminate the sheath and maximize the working space: sheathless transradial guiding catheters. Catheter Cardiovasc Interv. (2015) 86(1):59–60. 10.1002/ccd.26048 26014089

[12] Byrne RA Rossello X Coughlan JJ Barbato E Berry C Chieffo A. 2023 Esc guidelines for the management of acute coronary syndromes. Eur Heart J. (2023) 44(38):3720–3826. 10.1093/eurheartj/ehad191 37622654

